# Biotin Protein Ligase Is a Target for New Antibacterials

**DOI:** 10.3390/antibiotics5030026

**Published:** 2016-07-25

**Authors:** Jiage Feng, Ashleigh S. Paparella, Grant W. Booker, Steven W. Polyak, Andrew D. Abell

**Affiliations:** 1 Department of Chemistry, University of Adelaide, North Tce, Adelaide, SA 5005, Australia; dovekey_feng@hotmail.com; 2 School of Biological Sciences, University of Adelaide, North Tce, Adelaide, SA 5005, Australia; ashleigh.paparella@adelaide.edu.au (A.S.P.); grant.booker@adelaide.edu.au (G.W.B.); 3 Centre for Nanoscale BioPhotonics (CNBP), University of Adelaide, Adelaide, SA 5005, Australia

**Keywords:** antibiotic, biotin, biotin protein ligase, *Staphylococcus aureus*, inhibitor design, X-ray crystallography, in situ click chemistry

## Abstract

There is a desperate need for novel antibiotic classes to combat the rise of drug resistant pathogenic bacteria, such as *Staphylococcus aureus*. Inhibitors of the essential metabolic enzyme biotin protein ligase (BPL) represent a promising drug target for new antibacterials. Structural and biochemical studies on the BPL from *S. aureus* have paved the way for the design and development of new antibacterial chemotherapeutics. BPL employs an ordered ligand binding mechanism for the synthesis of the reaction intermediate biotinyl-5′-AMP from substrates biotin and ATP. Here we review the structure and catalytic mechanism of the target enzyme, along with an overview of chemical analogues of biotin and biotinyl-5′-AMP as BPL inhibitors reported to date. Of particular promise are studies to replace the labile phosphoroanhydride linker present in biotinyl-5′-AMP with alternative bioisosteres. A novel in situ click approach using a mutant of *S. aureus* BPL as a template for the synthesis of triazole-based inhibitors is also presented. These approaches can be widely applied to BPLs from other bacteria, as well as other closely related metabolic enzymes and antibacterial drug targets.

## 1. Introduction

Infectious diseases caused by pathogenic bacteria, such as *Staphylococcus aureus*, are a major threat to human health. The spread of antibiotic resistant strains, such as methicillin resistant *S. aureus* (MRSA), is particularly problematic with resistance having been developed to most penicillin-based antibiotics [1,2]. Antibiotic resistance arises in two major subsets of MRSA, hospital acquired MRSA and community acquired MRSA. Both have been described over the past decade in the USA [3], UK [4] and Australia amongst other countries [5]. The impact of MRSA is overwhelming, as these infections are more difficult to treat with increased associated healthcare costs. In the USA alone, the cost to treat hospital acquired-MRSA stands at $USD 9.7 billion annually [6], and community acquired-MRSA accounts for 18% of all MRSA incidents [3]. Overall, these factors have contributed to an increase in the mortality rate due to MRSA infections worldwide [7]. One critical strategy to combat drug resistant *S. aureus* is to develop new classes of antibacterials that are not subject to pre-existing resistance mechanisms [8]. This review presents biotin protein ligase (BPL) as a novel drug target, and discusses the design of small molecule inhibitors for antibacterial discovery.

## 2. Biotin Protein Ligase as a Novel Antibacterial Target

BPL, a vital enzyme present in all organisms, is responsible for the post-translational attachment of biotin 1 onto a specific lysine residue present in the active site of biotin-dependent enzymes, as shown in Scheme I [9,10]. *S. aureus* expresses two such enzymes, namely acetyl CoA carboxylase (ACC) [11] and pyruvate carboxylase (PC) [12], which are known to catalyze key reactions in important metabolic pathways. ACC is a critical enzyme for the carboxylation of acetyl-CoA to malonyl-CoA in fatty acid biosynthesis that is essential for cell membrane biogenesis and maintenance [13]. Biotin-activated PC is involved in the conversion of pyruvate to oxaloacetate required in the citric acid cycle that is central to a number of key metabolic pathways, such as gluconeogenesis and amino acid biosynthesis [14]. These metabolic pathways are essential for the survival and virulence of *S. aureus*, and, as such, BPL presents as an attractive drug target for new antibacterial drugs. Moreover, genetic knockout studies on various bacteria, including *S. aureus* [15,16], abolished cell growth in the absence of the *bpl* gene, highlighting that an alternative pathway for protein biotinylation does not exist in bacteria.

BPL acts as a transcriptional repressor [17,18,19] in addition to its pivotal role in the activation of ACC and PC. In the absence of non-biotinylated biotin-dependent enzymes, *S. aureus* BPL (*Sa*BPL) can form a dimer that is responsive to DNA binding. The *Sa*BPL dimer is a transcriptional repressor that controls the uptake and biosynthesis of biotin by binding specific DNA sequences in the promoters of the genes encoding these proteins. Therefore, BPL not only utilizes biotin, but it is also the key regulator that co-ordinates the import and synthesis of biotin in response to cellular demand. This bifunctionality makes BPL an attractive drug target as *S. aureus* is unlikely to readily develop target based resistance through mutation due to the intimate role played by BPL in multiple metabolic pathways [19].

## 3. Mechanism of Protein Biotinylation

**Figure 1:**
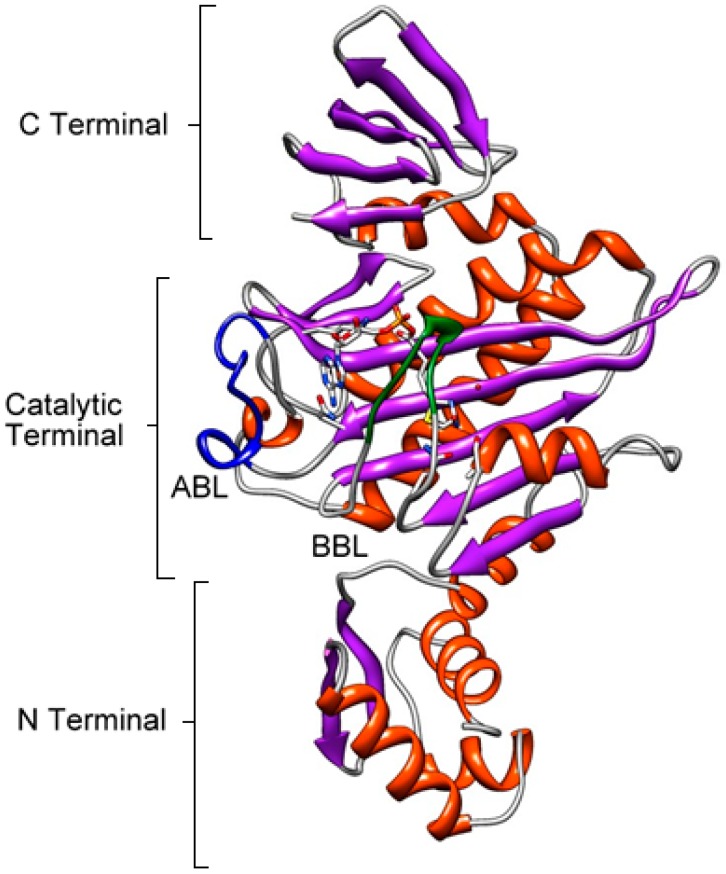
3D depiction of *Sa*BPL with biotinyl-5′-AMP 3 bound (PDB: 3V8L). The β sheets are shown in purple, α helices in orange, biotin-binding loop in green and ATP-binding loop in blue.

BPL catalyzes protein biotinylation through a two-step reaction mechanism, as shown in Scheme I [10,20]. In the first step, BPL catalyzes a condensation reaction between biotin 1 and ATP 2 to form the reaction intermediate biotinyl-5′-AMP 3, with the release of pyrophosphate (PPi). During this first step, biotin 1 binds to the biotin-binding pocket in BPL, which induces ordering of a biotin-binding loop within the enzyme (Figure 1). This disordered-to-ordered conformational change positions a key tryptophan residue (Trp127) in the active site, thereby creating the nucleotide binding pocket that facilitates binding of ATP 2. Reaction of biotin with the α-phosphate of ATP then produces the intermediate biotinyl-5′-AMP 3 to complete the first partial reaction. The complex of BPL with biotinyl-5′-AMP 3 then forms a protein: protein interaction with the unliganded biotin-dependent enzyme (i.e., the apo enzyme in Figure 1) to allow biotinyl transfer in the second partial reaction. During this final step, biotin is attached to the ɛ-amino group of the target lysine residue present in apo protein substrate to afford biotinylated ACC or PC (4, i.e., the holo enzyme in Figure 1), with the release of AMP. This reaction mechanism is conserved in all species, suggesting a high degree of homology amongst different BPL enzymes [21,22,23].

## 4. BPL Structure

**Figure 2:**
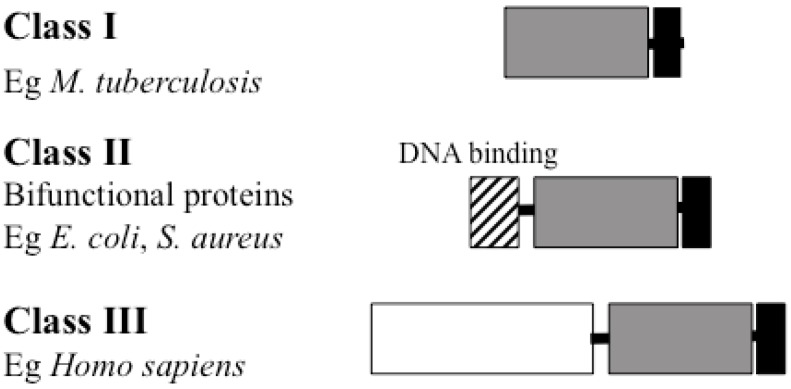
Schematic diagram of the three classes of BPL. The conserved catalytic (grey) and C-terminal domains (black) are highlighted. The relative sizes of the N-terminal extensions on class II and class III BPLs are represented.

BPL can be divided into three distinct structural classes, as depicted in Figure 2. Classes I and II include BPLs from Archaea, prokaryotes and plants. Class III contains BPLs from yeast, insects and mammals. All three classes of BPLs contain a conserved catalytic domain and a C-terminal cap domain that are essential for protein biotinylation [21]. Class I BPLs consists solely of the conserved catalytic and C-terminal domains. Class II BPLs contain an additional N-terminal domain that facilitates binding to DNA in the regulation of cellular uptake of biotin and biosynthesis of biotin, as described above. Class III BPLs have a larger N-terminal extension that is distinct from the DNA-binding domain of class II enzymes [24,25,26,27]. X-ray crystal structures of class I BPLs from *Mycobacteria tuberculosis* [28], *Aquifex aeolicus* [29] and *Pyrococcus horikoshii* [10], and class II BPL from *S. aureus* [30] and *Escherichia coli* [20] have been reported. An examination of these structural data reveals that all BPLs adopt a highly conserved protein fold within the catalytic domain. Of particular note are the biotin-binding loop (highlighted in green in Figure 1) and an ATP-binding loop (highlighted by blue ribbons in Figure 1) that are responsible for the ordered binding mechanism described above. A closer examination of these structural features is detailed below, with a view to designing inhibitors that can occupy the BPL active site.

### Catalytic Domain

**Figure 3:**
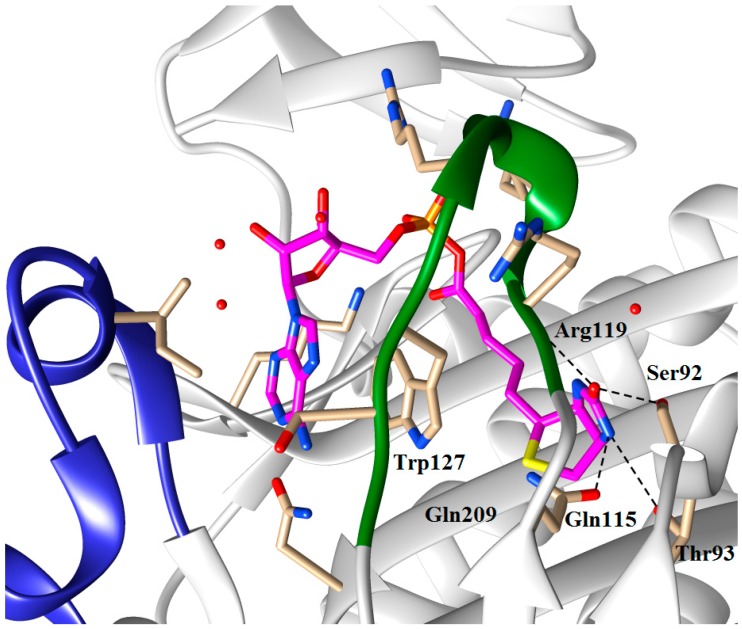
3D depiction of reaction intermediate biotinyl-5′-AMP 3 (highlighted in magenta) bound to *Sa*BPL (PDB: 3V8L) with a close examination of the biotin-binding pocket. Green ribbon highlights the biotin-binding loop and black dashes indicating hydrogen-bonding interactions between the ureido ring of biotin and *Sa*BPL.

As mentioned above, the catalytic domain of BPL contains two major ligand-binding pockets, one for biotin and the other ATP. The biotin-binding site consists of two distinct regions, a hydrophobic wall to accommodate the valeric acid chain on biotin and a glycine rich hydrophilic pocket to accommodate the heterocycle of biotin. Multiple hydrogen bonds are formed between the ureido ring of biotin and amino acid residues in the hydrophilic region of *Sa*BPL [31], namely Ser92, Thr93, Gln115 and Arg119, as depicted in Figure 3. These residues are highly conserved in BPLs from all species [32]. Biotin forms additional hydrophobic interactions with Trp127 and Gly209. The carbon chain on the valeric acid tail of biotin is orientated to a hydrophobic tunnel consisting of Gly118, Gly209, Gly188, Leu191 and Ile208 by induced fit binding of biotin in the first catalytic step of BPL [21,22]. The biotin-binding loop is believed to enclose the active site, thereby preventing the dissociation of ligand from the active site. Structural analysis with all the available crystal structures reveals a high degree of conservation in the biotin-binding pocket, as highlighted in Figure 3.

**Figure 4:**
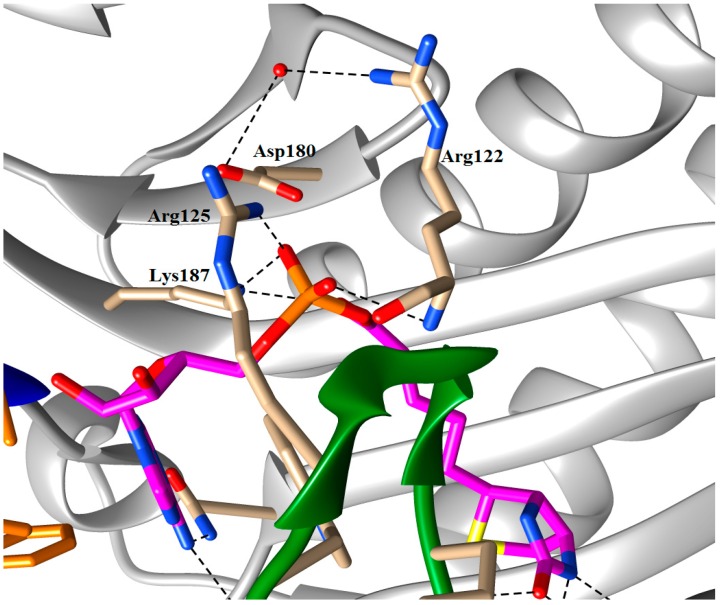
3D depiction of reaction intermediate biotinyl-5′-AMP 3 (highlighted in magenta) bound to *Sa*BPL (PDB: 3V8L) with a close examination of the phosphate-binding pocket. The biotin-binding loop is shown in green.

A highly conserved phosphate-binding domain is located between the biotin and ATP binding pockets of *Sa*BPL. A number of hydrogen bonds are observed between the phosphoroanhydride linker of biotinyl-5′-AMP 3 and the side chains of Lys187 and Arg125, as well as the backbone of Arg122 (Figure 4) [31]. The conserved “Gly-Arg-Gly-Arg^122^-X” motif present in the biotin-binding loop is critical in stabilizing binding of biotinyl-5′-AMP 3 by shielding it from solvent [20]. Of particular note is Arg122, which is central to a complex network of water-mediated hydrogen bonds with the side chain of Asp180 (Figure 4) [31,32]. This observation is also supported by studies with *E. coli* BPL where a point mutation of the equivalent residue (Arg118) results in dissociation rates enhanced by 100-fold for biotin 1 and 400-fold for biotinyl-5′-AMP 3 [32]. Mutation of Arg122 to glycine results in a “leaky phenotype” that has been exploited using in situ click chemistry to develop BPL inhibitors (described later) [33].

**Figure 5:**
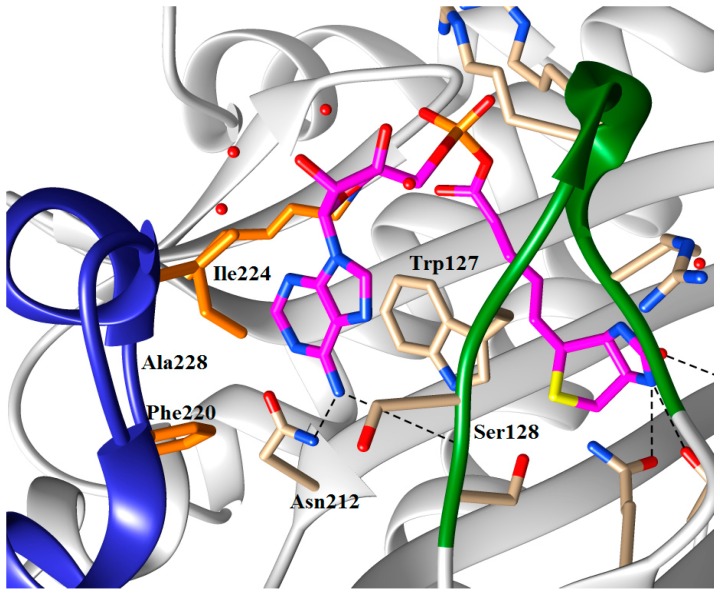
3D depiction of reaction intermediate biotinyl-5′-AMP 3 (highlighted in magenta) bound to *Sa*BPL (PDB: 3V8L) with a close examination of the ATP-binding pocket. Green ribbon highlighting the biotin-binding loop and blue ribbon highlighting the ATP-binding loop [31].

The previously mentioned, induced-fit binding of the biotin-binding loop (highlighted in green as in Figure 4) orientates the side chain of Trp127 of *Sa*BPL such that it creates a binding pocket for nucleotide binding. This binding is stabilized by a displaced parallel π interaction between the adenine ring of ATP and the indole ring of Trp127 [32,34]. It is noteworthy that this key binding interaction does not occur in the absence of biotin [10]. A number of hydrogen bonds are also observed between the adenine ring of ATP and Asn212 and Ser128 at the base of the ATP-binding pocket (Figure 5). Following ATP binding, the previously discussed condensation reaction between biotin and ATP occurs to give biotinyl-5′-AMP 3 [31]. The reaction intermediate adopts a distinctive “U-shaped” geometry so the biotinyl and adenyl moieties can bind in their respective binding pockets. The ATP-binding loop (highlighted in blue in Figure 5) helps to stabilize the reaction intermediate (3) via a disordered-to-ordered conformational change. Here, the ATP-binding loop folds over the adenosine moiety of 3 with associated hydrophobic interactions with 3 through Ile224, Ala228 and Phe220. This structural data provides a molecular explanation for the ordered ligand binding mechanism that is critical for the design of inhibitors.

## 5. BPL Inhibitors as New Antibacterials

*Sa*BPL is an attractive novel target for antibacterial development for three main reasons. Firstly, *Sa*BPL is the sole enzyme responsible for the biotinylation, and subsequent activation, of ACC and PC of *S. aureus* [35]. As these biotin-dependent enzymes play key roles in metabolic pathways critical for survival and virulence, BPL is an essential enzyme. Secondly, *Sa*BPL is the only enzyme in *S. aureus* responsible for regulation of biotin biosynthesis and import on cellular demand. Thus, targeting *Sa*BPL targets both the source of biotin and its utilization [17,18]. Thirdly, *Sa*BPL is not the target of any antibacterial currently in clinical use, thereby providing a novel mechanism of action. Recent structure-guided approaches to the design of small molecule inhibitors against *Sa*BPL have led to the discovery of BPL inhibitors that bind selectively to bacterial BPL over the human homolog. These studies are described further below.

### 5.1. Biotin Analogues as Antibacterial Agents

**Figure 6:**
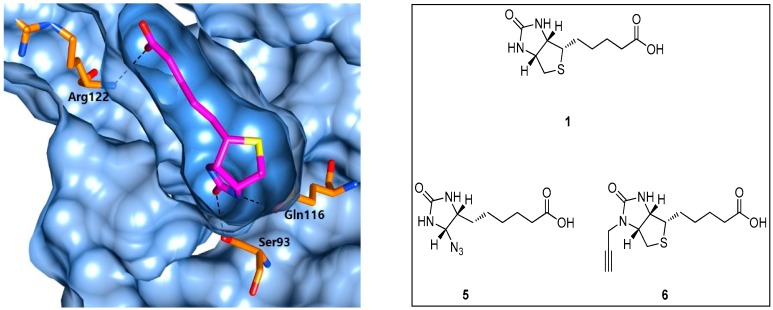
3D depiction of biotin 1 (highlighted in magenta) bound to *Sa*BPL (PDB: 3V8K) with a side view of biotin binding pocket (**left**); Chemical structures of biotin 1 and its analogues 5 and 6 (**right**).

An effective approach to disrupt protein biotinylation is to design small molecule inhibitors that bind tightly and specifically to the active site of bacterial BPL, thereby blocking all protein biotinylation. For example, modification to the ureido and thiophene ring of biotin gave rise to analogues 5 and 6 as shown in Figure 6 [31]. The study revealed that BPLs were highly specific to the natural structure of biotin 1 and did not utilize other biotin analogues as substrates [31]. In addition, amino acid sequence alignments highlight that the biotin-binding pocket is highly conserved amongst BPLs from all species, including human [30,31,36]. This presents a challenge for the design of selective inhibitors that target bacterial BPLs over the human equivalent. Moreover, analysis of available X-ray structures reveals a relatively small biotin-binding pocket for *Sa*BPL, thereby restricting opportunities to chemically modify the heterocycles of biotin 1.

Specific chemical modification of the carboxyl group of biotin 1 gave rise to a series of BPL inhibitors 7–13 as shown in Table 1 [31]. These compounds contain a hydroxyl, alkane and alkyne in place of the carboxyl group of biotin 1. The alcohol derivative 7 was found to be equally active against *Sa*BPL and *E. coli* BPLs with a *K_i_* between 3 and 4 μM. However, 7 displayed limited selectivity with only a 2.6-fold difference in *K_i_* for *H. sapiens* BPL (*Hs*BPL) versus *Sa*BPL (*K_i_* ≈ 9.0 μM) [31]. The more hydrophobic analogues 9–13 were more selective, e.g., 10 displayed approximately 12-fold selectivity for *Sa*BPL compared to *Hs*BPL [31]. An X-ray structure of *Sa*BPL in complex with biotin alkene 12 revealed a key hydrophobic interaction between the terminal carbon on the ligand and the side-chain of Trp127. Interestingly, increasing the length of the alkyl chain by a single carbon as in 13, resulted in reduced potency, presumably due to the disruption of this key bonding interaction [31]. Overall, this study suggests that biotin derivatives with chemical modifications at the biotin heterocycles and the valeric acid moiety are not ideal for achieving optimal potency and selectivity towards *Sa*BPL. However, derivatives 11 and 12 do provide an important starting point for further inhibitor development as discussed in section 5.3 below.

### 5.2. BPL Reaction Intermediate Analogues as Antibacterial Agents

**Figure 7:**
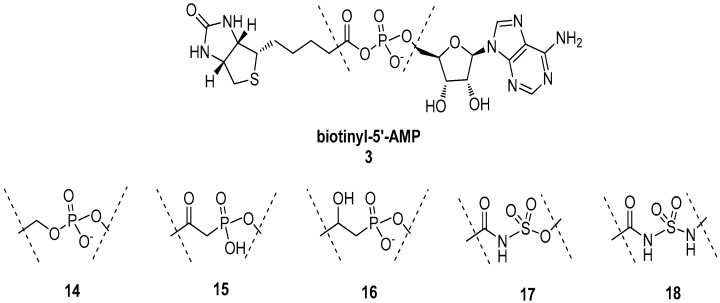
Reaction intermediate biotinyl-5′-AMP 3 and its mimics, biotinol-5′-AMP 14, β-ketophosphonate 15, β-hydroxyphosphonate 16, acylsulfamate 17, and acylsulfonamide 18.

As discussed earlier, the first step of the BPL reaction yields biotinyl-5′-AMP 3. The acyl phosphate group of 3 can be replaced with the non-hydrolysable and enzymatically stable phosphodiester bioisostere as in biotinol-5′-AMP 14 (Figure 7, below) [37]. Biotinol-5′-AMP 14 proved to be a potent inhibitor of *Sa*BPL (*K_i_* = 0.03 μM) while critically also possessing anti-*S. aureus* activity with a minimal inhibitory concentration of 1–8 μg/mL [38]. However, progressing biotinol-5′-AMP 14 as drug candidate is limited by its activity against *Hs*BPL with *K_i_* = 0.42 μM and also difficulty of synthesis. Two other phosphonate-based isosteres, as in 15 and 16, respectively, were initially developed by Sittiwong et al. for investigation of *Hs*BPL [39]. However, the β-ketophosphonate 15 and β-hydroxyphosphonate 16 analogues both showed reduced activity (*IC*_50_ of 39.7 μM and 203.7 μM, respectively) against *Hs*BPL compared to biotinol-5′-AMP 14 (*IC*_50_ = 7 μM against human BPL) [39].

Brown and co workers [37,40] described a sulfamoyl analogue 17 as a mimetic of the natural reaction intermediate 3 (Figure 7). However, this analogue rapidly decomposes and was thus difficult to assay [28,37]. A recent study identified sulfonamide analogue 18 as having improved stability compared to the sulfamoyl analogue 17 [28]. Significantly, 18 is a competitive inhibitor against biotin when assayed against *M. tuberculosis* BPL with an *IC*_50_ of 135 nM [28]. This analogue also displayed anti-mycobacterial activity against the virulent *M. tuberculosis* strain H37Rv as well as a number of multi-drug resistant *M. tuberculosis* strains, with a minimal inhibitory concentration ranging from 0.625 to 0.16 μM [28]. However, cytotoxicity was observed in the mammalian Vero cell line suggesting potential issue with selectivity for the bacterial BPL over the human homolog.

### 5.3. 1,2,3-Triazole Based Analogues

**Figure 8:**
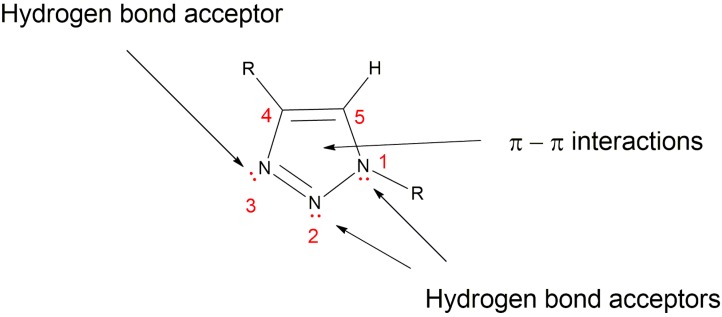
The assignment of 1,2,3-triazole with the potential intermolecular interaction sites.

Soares da Costa et al. identified a 1,2,3-triazole as a new and effective bioisostere for the labile phosphoroanhydride linker of biotinyl-5′-AMP 3 [41]. A triazole offers a number of advantages over the natural phosphate linker of 3. It is stable to acid/base hydrolysis, reductive and oxidative conditions, as well as typical physiologically conditions. This makes it resistant to metabolic degradation [42]. In addition, the 1,2,3-triazole motif has potential sites for hydrogen bonding (Figure 8), an ability to participate in π-π stacking interactions and it is easy to prepare.

**Figure 9:**
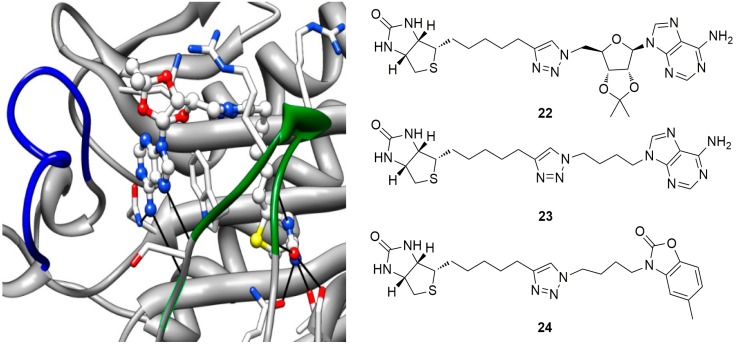
3D depiction of 1,4-triazole 22 bound to *Sa*BPL (PDB: 3V7C) (**left**); Chemical structures of 1,2,3-triazole analogues: 1,4-triazole 22 and 1,4-triazole 23 (**right**).

A series of 1,2,3-triazole based analogues of 20–23 (see Scheme II and Figure 9) was synthesized by alkyne azide cycloaddition (CuAAC) and these were tested for inhibitory activity against *S. aureus* and human BPLs [41]. In particular, reaction of biotin acetylene 12 and adenosine azide 19 gave the 1,4 disubstituted triazole 20 which displayed modest activity against *Sa*BPL (*K_i_* = 1.8 μM) but was effectively inactive against *Hs*BPL (*K_i_* > 33 μM) in vitro [41]. This was an important finding as it represented the first example of a BPL inhibitor with high selectivity for *S. aureus* BPL over the human equivalent. In addition, the 1,4-triazole 20 was not toxic against mammalian HepG2 cells in culture. Importantly, the 1,5-triazole regioisomer 21 (Scheme II) prepared via ruthenium alkyne azide cycloaddition (RuAAC) proved to be inactive against *Sa*BPL [41]. An X-ray crystallographic structure of *Sa*BPL in complex with 20 revealed that the 1,4-triazole provides the desired U-shape geometry on binding to *Sa*BPL, as observed for biotinyl-5′-AMP 3.

Based on the above assessment, a new generation of *Sa*BPL inhibitors was designed to target the ATP-binding site. A detailed analysis of the crystal structure of 20 bound to *Sa*BPL revealed an absence of hydrogen bonding between the ribose moiety of 20 and *Sa*BPL (Figure 9) [33,41]. Gratifyingly, the 1,4-triazole analogue 23, which lacks the ribose group, proved to be more potent than 20 against *Sa*BPL with *K_i_* of 0.7 μM [33] while retaining selectivity for the bacterial enzyme over the human homologue. Furthermore, the study also identified a biotin 1,4-triazole analogue containing a 2-benzoxalone group as a mimic of the adenine of 23 to target the ATP-binding pocket [41]. The 2-benzoxazolone containing 1,4-triazole 24 proved to be the most potent *Sa*BPL inhibitor with a *K_i_* = 0.09 μM [41]. Significantly, 24 exhibited >1100 fold selectivity for *Sa*BPL compared to human BPL. Thus, 24 represents the most potent and selective inhibitor of *Sa*BPL reported to date. Bacteriostatic activity was observed for 1,4-triazole 24 against *S. aureus* ATCC strain 49775, with the compound effectively reducing *S. aureus* cell growth by 80% at 8 μg/mL [41]. Both 1,4-triazoles 23 and 24 were not toxic in a cell culture model using HepG2 cells, highlighting these biotin triazoles as exciting hits for further antibiotic development [33,41].

## 6. In Situ Click Chemistry

In situ click chemistry has recently been reported as an alternative and more facile approach to optimize the biotin triazole series as inhibitors of *Sa*BPL [33,43,44,45]. Here, the target enzyme is used as a template to identify and bind optimum azide and acetylene fragments from a library of such structures. Once each azide and acetylene bind to their respective pockets, a cycloaddition reaction occurs in the absence of external catalysts (i.e., copper or ruthenium) to assemble the triazole. Moreover, as the biological target is actively involved in selecting its most potent inhibitor from a library of precursors, in situ click chemistry is able to circumvent the need to individually synthesize and screen all possible triazole combinations [46,47,48]. The BPL is only one of a select few examples of enzymes shown to catalyse the alkene azide cycloaddition reaction, as exemplified with biotin acetylene 12 and adenosine azide 19 in Scheme III [33]. This is possible because *Sa*BPL contains two well defined binding pockets, one capable of binding a biotin analogue and the other an adenine analogue, as revealed in the before mentioned X-ray structures.

An initial in situ click reaction was performed between biotin acetylene 12 (*K_i_* = 0.3 μM) and adenosine azide 19 using wild type *Sa*BPL as a template in an attempt to form 20 (Scheme III). Analysis of the reaction mixture by HPLC and mass spectrometry revealed 1.07 ± 0.1 mol of triazole 20 was formed per mol of *Sa*BPL [33]. The triazole formed by *Sa*BPL is presumed to be the 1,4-triazole 20 (*K_i_* = 1.18 μM), given that the 1,5-triazole 21 (see Scheme II) had earlier been shown to be inactive against *Sa*BPL [41]. An in situ experiment was then performed between biotin acetylene 12 and a small library of azides (25–28), as shown in Scheme III. The azide 19 was used as a reference, while 25–28 were designed to probe the importance of the furanose ring and also the length of the azide spacer with regards to potency. However, in this case triazole products were not detected by HPLC. This likely reflects a low turnover rate for the native BPL.

Attention was then focused upon improving the catalytic efficiency of the target enzyme, *Sa*BPL. The structural biology demonstrated that the before mentioned biotin-binding loop closes over the active site to prevent diffusion of the synthesized triazole from the active site and thereby preventing efficient turnover [33]. Of particular significance is Arg122, which is known to stabilize the “closed” conformation through a complex network of interactions with amino acid residues in the *Sa*BPL dimer interface and the C-terminal domain, as well as a water-mediated hydrogen bond with Asp180 [23,41]. It was proposed that an engineered variant of *Sa*BPL, with Arg122 substituted by glycine, would improve production of triazole 20 by increasing the enzyme’s turnover rate. This proposal is supported by studies with *Escherichia coli* BPL that have demonstrated that mutation of the equivalent residue (Arg118) results in enhanced dissociation rates for both biotin and biotinyl-5′-AMP [33].

A subsequent in situ click reaction of biotin alkyne 12 and azide 19 with *Sa*BPL-Arg122Gly gave vastly improved formation of triazole 20 (11.9 ± 0.7 moles per mol of enzyme) [33]. This clearly demonstrated that the mutant enzyme provides a template for cycloaddition, with an increased turnover rate compared to the wild-type enzyme. The “leaky mutant” thus provided much improved efficiency of reaction and sensitivity of detection. The library experiment using biotin alkyne 12 and azides 25–28 was repeated using the “leaky mutant”. Analysis of the product mixture by HPLC and LC/MS revealed efficient formation of 1,4-triazole 23 with a smaller quantity of a second 1,4-triazole 20 detected by MS but not HPLC. This observation was consistent with other multi-component in situ experiments where the higher affinity triazoles are formed to a greater extent. In our case, there is an overwhelming bias towards the formation of the more potent triazole 23 (*K_i_* = 0.66 ± 0.1 μM) over triazole 20 (*K_i_* = 1.83 ± 0.3 μM) [33,41]. None of the other possible triazole products were detected. This methodology provides a powerful tool for the identification of new inhibitors that target the BPL active site from libraries of precursor fragments. In addition, the study represents an important advance in in situ inhibitor optimization, where it was shown for the first time that a target enzyme can be engineered to improve efficiency and hence utility in such studies.

## 7. Future Directions

The emergence of bacteria resistant to chemotherapy is rendering our current arsenal of antibiotics less effective and in certain cases totally ineffectual. An important approach to address drug resistance is to develop new antibiotic classes that work through novel modes of action and that are not subject to existing resistance mechanisms. BPL inhibitors presents one such promising example as it is not the target of any chemotherapeutic currently in clinical use, thereby providing a novel mechanism of action. Importantly, we have identified a novel class of 1,2,3-triazole based BPL inhibitor (23 and 24) that (1) with a unique mode of antibacterial action; (2) unique selectivity for the bacterial BPL target over the human isozyme; (3) have antimicrobial activity against *S. aureus*; and (4) does not show toxicity either against a cultured human cell line or preliminary studies in rodent models. New analogues with improved solubility and/or new formulations are required to continue the development of lead compounds 23 and 24 towards pre-clinical antibacterial candidates.

The biotin triazole pharmacophore provides a valuable starting point for the chemical optimization of improved BPL inhibitors. X-ray analysis of lead compounds 23 and 24 bound to *Sa*BPL reveal that the adenosine and benzoxalone moieties bind into the nucleotide-binding pocket of the drug target. However, these approaches yield compounds with molecular masses greater than the 500 limit proposed as being optimum by Lipinski for drug-like candidates. One current strategy is to identify smaller structures that specifically bind into the ribose-binding pocket of *Sa*BPL. This latter approach with simplified structures is being pursued with the goal of optimizing simplified structures with greater drug-like properties and improved solubility. Moreover, we are extending our “smart” in situ click chemistry as an alternative approach to inhibitor optimization using our leaky mutant of the target enzyme (BPL) and new chemically diverse libraries of acetylene and azide coupling partners. These approaches provide valuable tools to aid in the development of new inhibitors against the BPLs of other clinically important bacteria and fungi. Furthermore, other enzymes that are targets for other diseases beyond antibacterial discovery can adopt the in situ guided approaches for inhibitor discovery we describe here for BPL. Particularly amendable to these methods are other ligases that synthesize an adenylated reaction intermediate from an organic acid and ATP, analogous to the BPL reaction mechanism. Of importance are ligases such as amino-acyl tRNA synthethases [49,50,51], bi-functional salicyl-AMP ligase [52,53], indole-3-acetic acid-amido synthetase [54] and pantothenate synthetase [55,56].

## Figures and Tables

**Scheme I:**
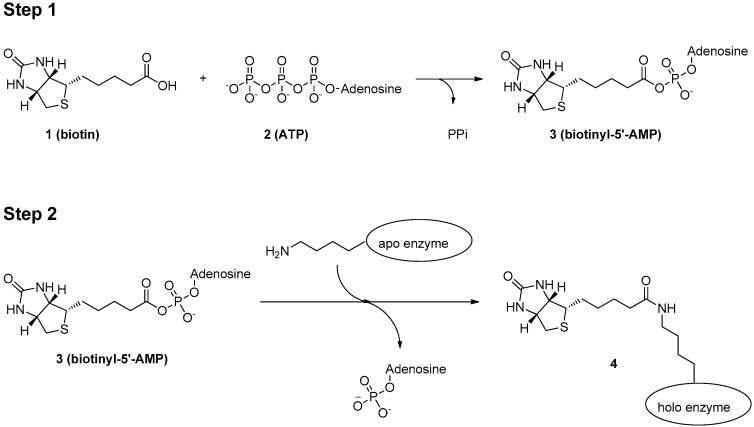
The catalytic mechanism of protein biotinylation catalyzed by biotin protein ligase (BPL).

**Scheme II:**
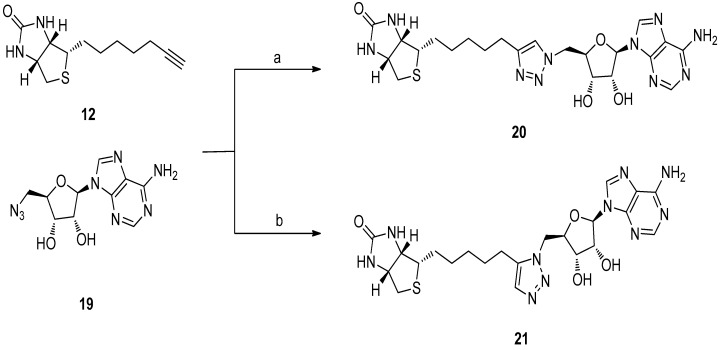
Synthesis of 1,4-triazole 20 and 1,5-triazole 21 from biotin acetylene 12 and azide 19. Conditions and reagents. (**a**) (i) copper nano powder, 2:1 AcCN/H_2_O, 4 h, sonication, 35 °C; (**b**) (i) Cp*RuCl(PPh_3_)_2_, 1:1 THF/DMF, 4 h, 70 °C.

**Scheme III:**
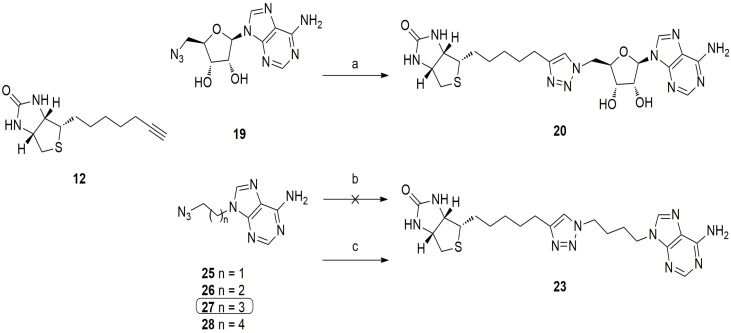
(**a**) In situ click reactions of acetylene 12 with azides 19 in the presence of wild type *Sa*BPL, 1,4-triazole 20 was confirmed by HPLC; (**b**) In situ click reactions of acetylene 12 with azides 25–28 in the presence of wild type *Sa*BPL, no triazole products were observed by HPLC; (**c**) In situ click reactions of acetylene 12 with azides 25–28 in the presence of *Sa*BPL Arg122-Gly, 1,4-triazole 23 was confirmed by HPLC.

**Table 1:** Biotin analogue series [30].

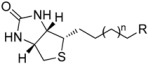	n	R	*Sa*BPL *K_i_* (μM)	*Ec*BPL *K_i_* (μM)	*Hs*BPL *K_i_* (μM)
**7**	2	OH	3.4	4.0	9.0
**8**	3	OH	>20	>20	>20
**9**	1	CH_3_	0.05	1.1	0.1
**10**	2	CH_3_	0.5	7.3	6.4
**11**	1	C≡C	0.08	0.9	0.2
**12**	2	C≡C	0.3	7.3	3.5
**13**	3	C≡C	2.4	20	12

## Abbreviations

The following abbreviations are used in this manuscript:

ACC	Acetyl CoA carboxylase
AMP	Adenosine-5′-monophosphate
ATP	Adenosine-5′-triphosphate
BPL	Biotin protein ligase
CuAAC	Copper mediated alkyne azide cycloaddition
HPLC	High-performance liquid chromatography
*Hs*BPL	*Homo sapiens* biotin protein ligase
*K_i_*	Inhibition constant
MRSA	Methicillin resistant *S. aureus*
PC	Pyruvate carboxylase
RuAAC	Ruthenium mediated Alkyne Azide cycloaddition
*Sa*BPL	*Staphylococcus aureus* biotin protein ligase
